# Validity and reliability of resiliency measures trialled for the evaluation of a preventative Resilience-promoting social-emotional curriculum for remote Aboriginal school students

**DOI:** 10.1371/journal.pone.0262406

**Published:** 2022-01-11

**Authors:** Gary Robinson, Eunro Lee, Bernard Leckning, Sven Silburn, Tricia Nagel, Richard Midford

**Affiliations:** 1 Centre for Child Development and Education, Menzies School of Health Research, Charles Darwin University, Casuarina, Australia; 2 School of Health & Biomedical Sciences, College of STEM, RMIT University, Melbourne, Australia; 3 Wellbeing and Preventable Chronic Diseases Division, Menzies School of Health Research, Charles Darwin University, Darwin, Australia; Temple University, UNITED STATES

## Abstract

**Purpose:**

We aimed to test the reliability and validity of two brief measures of resilience adopted for the evaluation of a preventative social-emotional curriculum implemented for Aboriginal middle school students from socially disadvantaged remote communities in Australia’s Northern Territory. The questionnaires chosen were intended to measure psychological resilience and socio-cultural resilience as complementary dimensions of the capacity to cope in circumstances of significant life stress and risk of self-harm.

**Methods:**

Confirmatory factor analysis (CFA) was conducted to assess construct validity of the 10-item Connor-Davidson Resilience Scale (CD-RISC-10), a measure of psychological resilience, and the 12-item Child and Youth Resilience Measure (CYRM-12), a measure of socio-cultural resilience, with a sample of 520 students. Associations between resilience and psychological distress and emotional and behavioural difficulty were analysed in relation to life stressors to assess criterion validity of the scales.

**Results:**

CFA provided support for the validity of the respective constructs. There was good fit for both scales. However, assessment of criterion validity of the scales suggested that the adapted measure of socio-cultural resilience (CYRM-12NT) showed higher reliability and a clearer indication of predictive validity than the measure of psychological resilience (CD-RISC-10).

**Conclusions:**

The CYRM-12NT appears to be a more useful measure of resilience among Aboriginal youth exposed to significant life stress and disadvantage. However, both measures may require further development to enhance their validity and utility among potentially at-risk adolescents in socially, culturally and linguistically diverse remote Aboriginal communities.

## Introduction

Young Aboriginal people in remote communities are subject to multiple sources of risk and disadvantage from early childhood. Rates of child protection intervention and subsequent rates of incarceration, self-harm and suicide during adolescence in Aboriginal communities are among the highest in Australia [1,2]. Suicide is the leading cause of death for Aboriginal adolescents, and rates in remote communities are 2–3 times higher than in major centres [3]. A high proportion of remote children are developmentally vulnerable at school entry and low rates of school attendance and of completion of secondary education point to significant educational disadvantage [4]. Furthermore, many remote Aboriginal communities have been characterized as ‘communities of risk’ in which residents are frequently exposed to stressors associated with exposure to drinking, peer and family violence and suicidal behaviour [5,6].

Strategies to promote youth resilience both through universal, school-based programs and through selective or indicated interventions increasingly form part of suicide prevention programs [7]. However, few if any resilience-building programs have been made available for remote Australian Aboriginal youth. The present study presents findings from one of a small number of recent projects aiming to moderate risks associated with psychological distress and exposure to suicidal behaviour among Aboriginal youth [8].

Remote communities in the Northern Territory consist of linguistically, socially and culturally diverse populations. Most residents speak one or more Aboriginal languages and are descendants of traditional landowning groups who, despite significant disruption of family patterns and social organization, continue to practice traditional customs and to maintain extended family relationships based on systems of kinship and associated beliefs. For young people, these social and cultural frameworks strongly influence social-emotional learning and resilient adaptation.

The Skills for Life (SFL) program is a curriculum intended for Aboriginal students in middle school (grades 7–9) in remote communities. It was developed in collaboration with teaching staff, community leaders and youth workers at a secondary college in a remote region of the Northern Territory of Australia from 2013–2015 [9]. Following established models for school-based social and emotional learning, the 12—week SFL curriculum aims to build emotional self-awareness, empathy and capacity for self-regulation, and to strengthen relationships, social problem-solving and help-seeking within a culturally informed framework for social and emotional wellbeing [10]. It seeks to promote resilience in the face of challenges commonly faced by young people in remote communities. After a preliminary trial, SFL was implemented in four schools located in remote communities each with wholly Aboriginal student enrolments and in one boarding school attended by remote Aboriginal students located in the capital city, over a three-year period from 2016–2018. Publication of results of the evaluation of program outcomes is forthcoming.

### Measuring resilience in contexts of risk and disadvantage

A methodological review concluded that there was no ‘gold standard’ among measures of resilience and recommended that attention be given to publication of validation data when measures are used [11]. This study presents a detailed examination of the validity of two measures chosen to measure both psychological and socio-cultural resilience for the evaluation of SFL.

Resilience is widely understood as the capacity to adapt and flourish in the face of adversity or significant trauma [12]. In explaining resilience, positive psychology has emphasized the protective influence of “individual competencies, resources, and psychological strengths” [13]. Individual strengths such as optimism and confidence in ability to cope may protect against psycho-social risk including suicidality [14–16]. In this tradition, school-based programs to build individual resilience and reduce psychological symptoms have been widely implemented [17]. By contrast, social-ecological theories of resilience have emphasized that resilience is a function of the resources made available through families and communities [18]. Ungar argues that, “Aspects of a community’s social and physical ecology are more important to the resilience of its members than the qualities of individuals alone” and that the dependence of individuals on external social resources is greater for those who are disadvantaged and at risk [19]. In this view, strategies to build resilience among young people should focus on building awareness of and capacity to negotiate access to social relationships and external supports.

Surveys to assess the resilience and wellbeing of children and youth frequently distinguish internal resources (strengths, competencies, dispositions) from external resources (relationships, supports, connectedness) [20]. A recent review of measures of resilience for Indigenous adolescents in Canada, Australia, New Zealand and the United States identified three constructs: Individual Assets, Environmental Resources and Cultural Resilience [21]. Other research suggests that external–environmental, relational, socio-cultural–resources interact with individual resilience in complex ways to which any simplified dichotomy in measurement between internal and external resources is unlikely to do justice [18,22]. The presence or absence of single resources–even those as centrally important as parental support—do not determine resilient outcomes, but rather how resources interact with, facilitate access to, or limit each other in a given context. A recent multi-country study described adolescent resilience as a network of interacting resources within which the strength of associations between resources varies across communities and cultures, even where comparable indices of disadvantage and adversity may be observed [23]. These perspectives suggest that at a minimum both individual and socio-cultural dimensions must be incorporated in strategies to measure resilience.

### Aims

This study aims to examine the validity and reliability of two measures of *psychological resilience* and *socio-cultural resilience*, respectively, as completed by remote Aboriginal students enrolled in grades 7–10 in five middle and secondary schools who participated in the SFL program. To establish criterion validity of the measures, we test the association between students’ resilience scores and levels of psychological distress and emotional and behavioural difficulties when controlling for the number of student-reported life stressors. That is, we ask whether higher resilience moderates the impact of exposure to life stress on students’ psychological wellbeing.

## Methods

### Procedure

Procedures for informed consent were approved by the Human Research Ethics Committee of the Northern Territory Department of Health and the Menzies School of Health Research, (Approval no. 13–2120), the NT Department of Education and the principal of each school. Parents were contacted before commencement of the program in consultation with school engagement officers. Information about the program was provided, with explanation about the aims of the evaluation and the purposes of data gathered, informing parents of their right to “say no” to their child’s participation and procedures for doing so at any time. Of 542 parents informed about the program, 22 declined consent over three years.

### Data-gathering

Using a method previously piloted [24], students were withdrawn from class singly, in pairs or small groups for approximately 20 minutes for completion of questionnaires in English during an interview. Research assistants (graduate and postgraduate students or project officers) were trained in questionnaire administration and after a scripted introduction read aloud each item while students read a paper copy on which they indicated their response to each item. Only 5% of students were able to complete questionnaires independently.

### Measures

The *Connor-Davidson Resilience Scale (CD-RISC-10)* was chosen to measure individual (psychological) dimensions of resilience in response to difficulty, referred to here as “psychological resilience” [11,25]. Based on a 25-item scale, it has been extensively used in circumstances of recovery after clinical risk, illness, post-traumatic stress, warfare and disaster [26] and in a sample of children following injury [27]. The CD-RISC-10 negatively correlates with depression, anxiety and behaviour problems and has been found to measure characteristics such as persistence, optimism and confidence in ability to cope that may buffer against psycho-social risk including suicidality [14–16]. CD-RISC-10 scores in an adult community sample have been found to vary with age, sex and education levels, and with histories of child maltreatment [28]. The measure has been validated in a representative community sample of Australian adolescents and young adults (*N* = 1000) [29]. However, a recent study in an urban and rural sample of young Aboriginal adults (*N* = 110) did not confirm scale properties [30]. The scale consists of 10-items, including: “I can handle it when change happens”, “I don’t give up easily when I fail” and “Having to cope with stress can make me stronger”. Responses are recorded within a 5-point Likert-type scale ranging from 0 (Never true) to 4 (Always true) [25].

The *Child and Youth Resilience Measure (CYRM-12)* was chosen to assess participants’ access to external social and cultural resources, referred to here as “socio-cultural resilience”. It has been validated with both clinical and general samples [31] and is based on a longer scale, the CYRM-28, recently evaluated with an Australian Aboriginal boarding school population [11,32]. International studies have confirmed its utility and validity as a culturally and contextually sensitive measure of resilience, but have drawn attention to a possible lack of sensitivity to between-community differences [33,34]. The *CYRM-12* has 12 items including, “Getting an education is important to me”, “I know who or where I can go to in my community to get help” and “In my community I am able to learn skills and knowledge that will help me in life”. To strengthen information for the school sector, an item relating to education was added, “At my school I can learn skills that will help me get work”. One item proved consistently difficult to administer, **“**I am able to solve problems without harming myself or others**”** and was removed. The designation, CYRM-12NT denotes adaptation for the Northern Territory (NT) of Australia. Responses are recorded within a 5-point Likert-type scale ranging from 0 (Never true) to 4 (Always true) [31].

In addition, two measures of psychological wellbeing and psychosocial risk adopted for the evaluation project provided tests of criterion validity of the resilience measures.

*The Kessler 6 Mental Health Scale (K6)* is a measure of psychological distress widely used to screen for risk of mental illness [35]. Example items include, “About how often did you feel that everything was an effort, too hard?” and “About how often did you feel so sad or depressed that nothing could cheer you up?” Respondents answer each item on a 5-point scale ranging from 1 (None of the time) to 5 (All of the time).

*The Strong Kids Symptom Scale (SKS)* assesses symptoms of emotional and behavioural difficulty within the last four weeks. It was adapted from the Strong Kids Symptom Scale (Grades 3–8) and the Strong Teens Symptom Scale (Grades 9–12) developed by Merrell and associates for the evaluation of a social-emotional curriculum [36]. It includes items such as: “I can’t deal with my problems”; “I argue with other people”; “I get so mad I break things”; “I worry about things.” Respondents answer each item on a 5-point scale ranging from 0 (None of the time) to 4 (All of the time).

Scale scores for these measures were the sum of individual item scores. Higher scores indicated heightened levels of each construct, that is higher levels of resilience, psychological distress or emotional and behavioural difficulty.

Evidence suggests that exposure to current and past life stressors including both chronic adversity and acute adverse events may predict lower resilience and, potentially, suicidal behaviour [37,38]. As indicated, remote Aboriginal youth are exposed to multiple life stressors [5]. To assess exposure to life stress experienced by participating students in their home communities, a *Life Stressors Checklist* was developed. It was based initially on a review of relevant surveys and revised after consultations with knowledgeable community members, and subsequently confirmed using an adapted nominal group technique. Lists were compiled in group discussion and students were given stickers to put against their personal choices. In this way, a ranking of challenges faced by young people in their communities was established. The *Life Stressors Checklist* consists of 8 items answered dichotomously with either *Yes* or *No*. Seven items included the following: “I have been teased or bullied”; “Someone close to me has passed away”; “Someone close to me has tried to hurt themselves” and “There has been a lot of drinking where I am living”. An 8th item asked whether students had experienced any other stressor in the last four weeks with the option for explanation. Screening of approximately 20% of responses to this item suggested that there was a mix of experiences not covered by other items, such as “imprisonment of a family member” and partial restatement of other items, such as “someone close passed away” but with additional elements suggestive of acuity such as “after death of [grandparent], there has been constant family drinking and fighting”. Thus, despite some possible overlap with other items, there are grounds for retention of item 8. Item analysis showed that this item added to internal consistency of the scale.

### Participants

Participants were 520 remote Aboriginal students in grades 7–10 at five schools at first assessment before commencing the SFL program (mean age = 13.46; *SD* = 1.24, ranging 10–19 years; female = 50%). Numbers of students by mean age, sex and by school are presented in Table 1. Male and female sex were assigned by a single M/F option checked at interview and confirmed with school records.

**Table 1:** Sample characteristics by school.

	Total	School 1	School 2	School 3	School 4	School 5
*N* (Participants)	520	147	27	83	109	154
Females (%)	50.1	45.6	55.6	53.0	53.7	49.4
Age (Mean, years) (*SD*)	13.5 (1.24)	13.2 (0.90)	12.4 (0.72)	14.0 (1.15)	13.1 (0.86)	13.9 (1.55)
Range	10–19	11–15	11–14	12–17	11–15	10–19

The number of classrooms in which the curriculum was taught varied from one classroom in the smallest school to six classrooms in the largest. Participating classes were both co-educational and single sex and were variously streamed by the schools according to attendance, engagement and academic level. Some classes combined year-levels, such as years 7, 8 and 9, or 7 and 8.

### Analysis overview

Confirmatory factor analysis (CFA) was conducted for each of CD-RISC-10 and CYRM-12NT using Mplus 8 [39]. Because the Chi-Square statistic is sensitive to the sample size and underestimates the model fit for CFA analysis with a large sample, alternative model fit indices were also used. Model fit was judged as satisfactory when the Comparative Fit Index (CFI) and Tuker-Lewis Index (TLI) were close to or larger than 0.95; the cut-off for Root Mean Square Error of Approximation (RMSEA) was close to but less than 0.06, and for Standardized Root Mean Square Residual (SRMR) was 0.08, as recommended by Hu and Bentler [40,41].

Reliability analysis was conducted using SPSS V26. Cronbach’s alpha coefficients were estimated as tests of internal consistency for both measures and compared across schools. Analysis of variance (ANOVA) was used to establish initial associations between scores. Following zero-order correlation analysis, as a test of criterion validity, hierarchical multiple regression analysis was conducted to determine whether the two resilience measures predicted lower scores for psychological distress (K6) and emotional-behavioural difficulties (SKS) when controlling for self-reported life stressors. Following comparable studies of scale performance, age and sex are also controlled for in analysis [32].

## Results

### Construct validity

Data screening showed that measurements of both resilience scales were not normally distributed. The CD-RISC-10 showed various non-normal distribution shapes across the items (Kolmogorov-Smirnov test, *p* < .001 for all items), with some items showing both unexpected numbers of low and unexpected numbers of high scores. CYRM-12NT showed positive skew with large endorsement frequencies of the “All the time” response option (Kolmogorov-Smirnov test, *p* < .001 for all items). Accordingly, the MLR (Maximum Likelihood Robust to non-normal data) estimator was used in the CFA, while the bootstrapping method was used to estimate the confidence intervals in the ANOVA and regression analyses for validity and group comparison analyses using SPSS. In order to ensure confidence in the results, scale responses were also treated as categorical variables in supplementary analysis using a robust weighted least squares (WLS) estimator [42].

#### Construct validity: Confirmatory factor analysis of CD-RISC-10

As shown in Table 2, 9 of 10 items showed factor loadings larger than .3 and ranged from .28 to .42. The CFA model showed good fit of the hypothesised model with the data (*χ*^*2*^ *(35*, *N =* 520*)* = 49.63, *p* = .0517; *CFI* = .95, *TLI* = .93, *SRMR* = .03, *RMSEA* = .03, 90% CI: 0.000–0.045).

**Table 2:** Confirmatory factor analysis of the 10-item Connor-Davidson Resilience Scale (CD-RISC-10): Psychometric comparisons with Campbell-Sills & Stein, (2007) [25].

Item	Description	Factor Loading	
		Present sample	Previous study*
1	I can handle it when changes happen	.37	.44
2	I can deal with whatever comes my way	.37	.72
3	I try to see the funny side when problems come up	.28	.46
4	Having to cope with stress can make me stronger	.31	.58
5	I tend to bounce back quickly after illness, injury or hard time	.42	.61
6	I believe I can achieve my goals even when things stand in my way	.39	.63
7	I can stay focused and think clearly under pressure	.38	.62
8	I don’t give up easily when I fail	.40	.63
9	I think of myself as a strong person who can deal with difficulties and challenges in life	.39	.74
10	I can handle bad feelings like sadness, fear and anger	.40	.57
Determinacy		.79	.93
Scale Reliability		.62	.85
*M*		19.82	27.21
*SD*		6.72	5.84

*Note*. Factor loadings in the third column are from the present sample. In the fourth column are factor loadings from a previous study* by Campbell-Sills & Stein (2007) [25].

In supplementary analysis, when treating responses as ordered categorical variables, results were similar to the main model results, but slightly more strongly indicative of good model fit. Factor loadings using the RWLS estimation method ranged from .33 to .45 and were slightly higher than the MLR results (see S1 Table). Model fit indices were consistent with good fit, according to recommendations for this method [43].

#### Confirmatory factor analysis of CYRM-12NT

The CYRM with adjusted 12 items showed factor loadings from .34 to .56 that were all larger than the .3 criterion (Table 3). The overall model fit was satisfactory (*χ*^*2*^ (53, *N =* 520*)* = 100.93, *p* < .001; *CFI* = .93, *TLI* = .91, *SRMR* = .04, *RMSEA* = .04, 90% CI: 0.029–0.054).

**Table 3:** Confirmatory factor analysis of the 12-item Child and Youth Resilience Measure (CYRM-12NT): Psychometric comparisons with Liebenberg et al., 2013 [31].

Item	Description	Factor Loading	
		Present Study	Previous Study*
1	I have people who I look up to	.34	.99
2	Getting an education at school is important to me	.61	.62
3	My parents/caregivers know a lot about me	.46	.74
4	When I start things I always try to finish them	.37	.26
6	I know who or where I can go to in my community to get help	.44	.28
7	I feel that I belong at my school	.46	.83
8	My family will stand by me when I am having a hard time	.35	.82
9	I have friends who will stand by me when I am having a hard time	.42	.54
10	I am treated fairly in my community	.37	.55
11	In my community I am able to learn skills and knowledge that will help me in life	.52	.23
12	Culture in my family and community is important to me	.48	.75
13^a^	At my school I can learn skills that will help me get work	.56	NA
Determinacy		.87	NA
Scale Reliability		.75	.75
*M*		45.11	44.16
*SD*		8.44	4.53

*Note*. The third column lists factor loadings for the present sample. Item 13a is an item of CYRM-12NT that replaced item 5 of the CYRM-12 for the present study. The fourth column lists loadings reported for CYRM-12 in a previous study*. Means and standard deviations were calculated using version 1 and version 2 statistics in Table 2 on p. e133 (Liebenberg et al., 2013 [31]). NA: Not Available.

In supplementary analysis, when treating responses as ordered categorical variables, results were similar to the main model factor loadings and model fit indices and slightly more indicative of good fit. Factor loadings based on the RWLS estimation method ranged from .38 to .65 and were slightly higher than the MLR results. Model fit indices (*CFI* = .95, *TLI* = .94) were slightly better than in the main analysis (see S2 Table).

#### Reliability analysis

As a test of internal consistency, Cronbach’s alpha coefficients were calculated for each scale for the whole sample and by school (Table 4, below).

**Table 4:** Cronbach’s alpha, mean scores and standard deviations for all measures by school.

Measures	*Alpha/Mean*	All	School 1	School 2	School 3	School 4	School 5
CD-RISC-10	*Α*	.62	.67	.70	.53	.64	.57
	*M (SD)*	19.82 (6.72)	19.77 (7.22)	17.85 (7.50)	20.60 (5.89)	19.24 (6.65)	20.43 (6.64)
CYRM-12NT	*Α*	.75	.75	.68	.73	.65	.77
	*M (SD)*	45.11 (8.44)	44.01 (8.44)	47.33 (7.52)	48.78 (7.41)	46.88 (6.61)	42.53 (9.24)
K6	*Α*	.50	.51	.62	.54	.56	.39
	*M (SD)*	15.58 (4.43)	16.16 (4.54)	14.07 (4.79)	15.23 (4.49)	15.82 (4.35)	15.32 (4.22)
SKS	*Α*	.68	.72	.68	.71	.68	.63
	*M (SD)*	15.81 (6.89)	16.90 (7.33)	15.19 (7.31)	15.96 (6.84)	15.74 (6.54)	14.92 (6.63)

*Note*. CD-RISC-10: The Connor-Davidson Resilience Scale; CYRM-12NT: The Child and Youth Resilience Measure NT; K6: The Kessler 6 Mental Health Scale; SKS: Strong Kids Symptoms Scale.

*Reliability*: *CD-RISC-10*. The alpha coefficient of CD-RISC-10 was .62. Only four items, 5, 8, 9, and 10 showed item-total correlations larger than .3. For the five schools, alphas were .67, .70, .53, .64, and .57.

*Reliability*: *CYRM-12NT*. The internal consistency of the CYRM-12NT was acceptable (α = .75). The first item, “I have people to look up to” (*r* = .29) did not meet the .3 criterion for item-total correlations. The correlations for other items ranged from .33 to .49. For school samples, alphas were .75, .68, .73, .65 and .77.

Values for the K6 were well below acceptable levels at .5 overall, while, for the SKS, they were at acceptable levels. Internal consistency of the Life Stressors Checklist, as assessed by the Kuder-Richardson 20 (KR20) coefficient for dichotomous measures, was low at .51 and was positively skewed (skewness = .28, Standard Error = .107).

### Criterion validity

To test criterion validity of the resilience measures, hierarchical multiple regression models were used to test whether higher levels of resilience predicted lower student distress and emotional and behavioural difficulties after controlling for age, sex and self-reported life stressors. The distribution of reported stressors is described below. ANOVA was then used to describe the association between numbers of life stressors reported and levels of psychological distress and emotional-behavioural difficulties.

#### Controlling for life stressors

**Fig 1:**
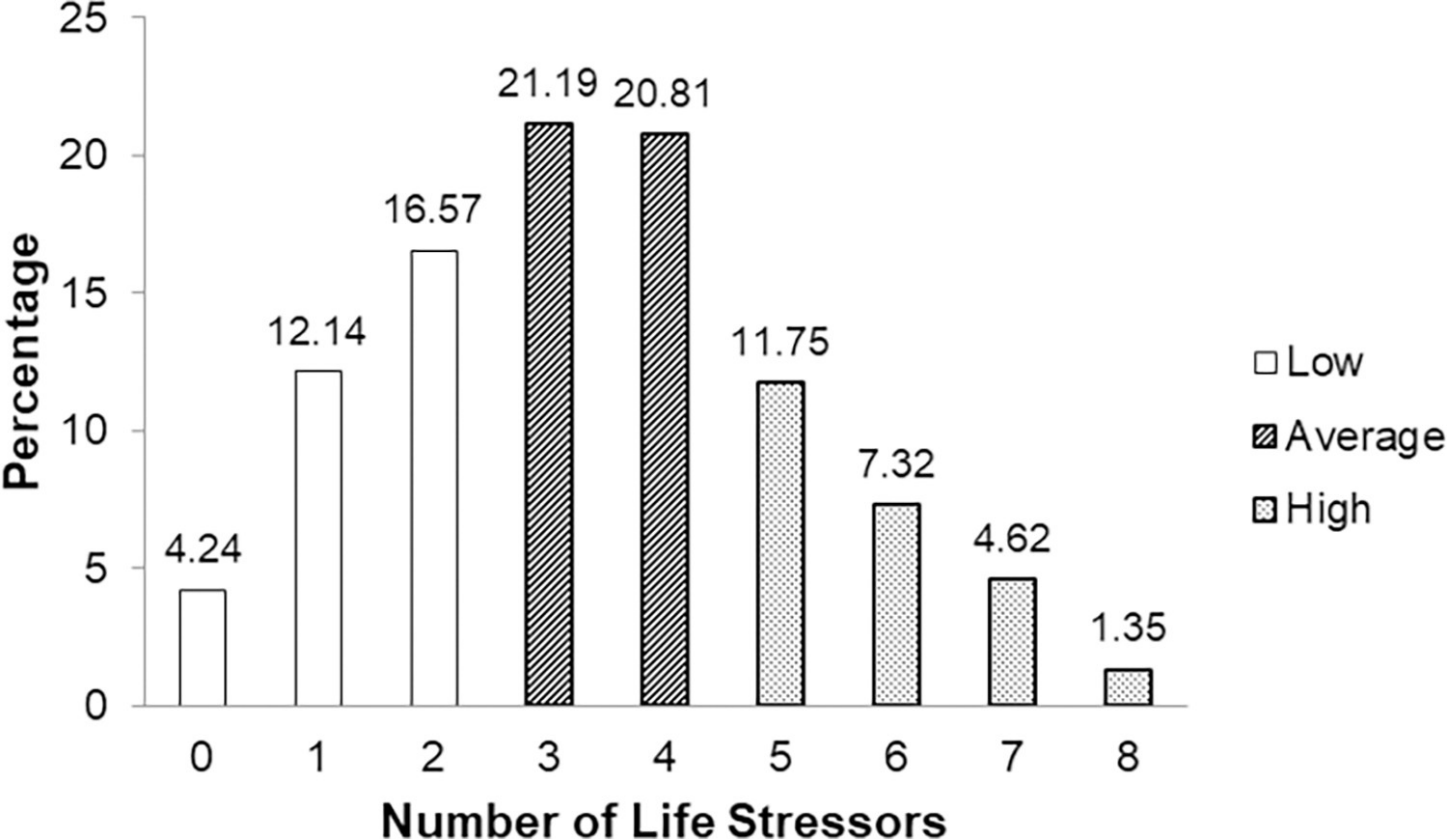
Frequency of reported life stressors for all schools: Life stressors checklist.

As shown in Fig 1, 95% of students reported at least one life stressor in the past four weeks, with on average above 3 life stressors reported for the whole sample (M = 3.38; SD = 1.82).

The average number of student-reported life stressors for each school ranged from just under 3 to 3.5. The analysis of variance (one-way ANOVA) between number of life stressors and both psychological distress (K6) and emotional and behavioural difficulties (SKS) scores resembled a graded relationship, with additional stressors indicating higher scores in each measure (see S3 Table). Following an approach adopted in other studies [44], for purposes of analysis and interpretation, three groups of reported life stressors, 0–2; 3–4 and 5–8 were used as comparison variables in analysis of the association between the resilience scores and both K6 and SKS scores. These groups are referred to as low (0–2); medium (3–4) and high (5–8) levels of life stress in the analysis.

Table 5 sets out correlations between study variables. Due to violation of the normality assumption, the bootstrapping technique was used to estimate confidence intervals of the coefficients.

**Table 5:** Correlations between study variables.

Measures	1	2	3	4	5	6	7
1.CD-RISC-10	-						
2.CYRM-12NT	.44**	-					
3.K6	16**	-.04	-				
4.SKS	.21**	0	.63**	-			
5.LS	22**	.08	.28**	.40**	-		
6.Age	.02	-.04	-.02	-.04	-.09*	-	
7.Sex	-.05	.02	-.02	.02	-.07	.02	-

*Note*: *N = 518–520*. Males were coded as 0; females were coded as 1.

*p < .05.

**p < .01 (2-tailed).

CD-RISC-10: The Connor-Davidson Resilience Scale; CYRM-12NT: The Child and Youth Resilience Measure NT; K6: The Kessler 6 Mental Health Scale; SKS: The Strong Kids Symptoms Scale; LS: The Life Stressors Checklist.

#### Levels of resilience according to level of psycho-social risk

**Fig 2:**
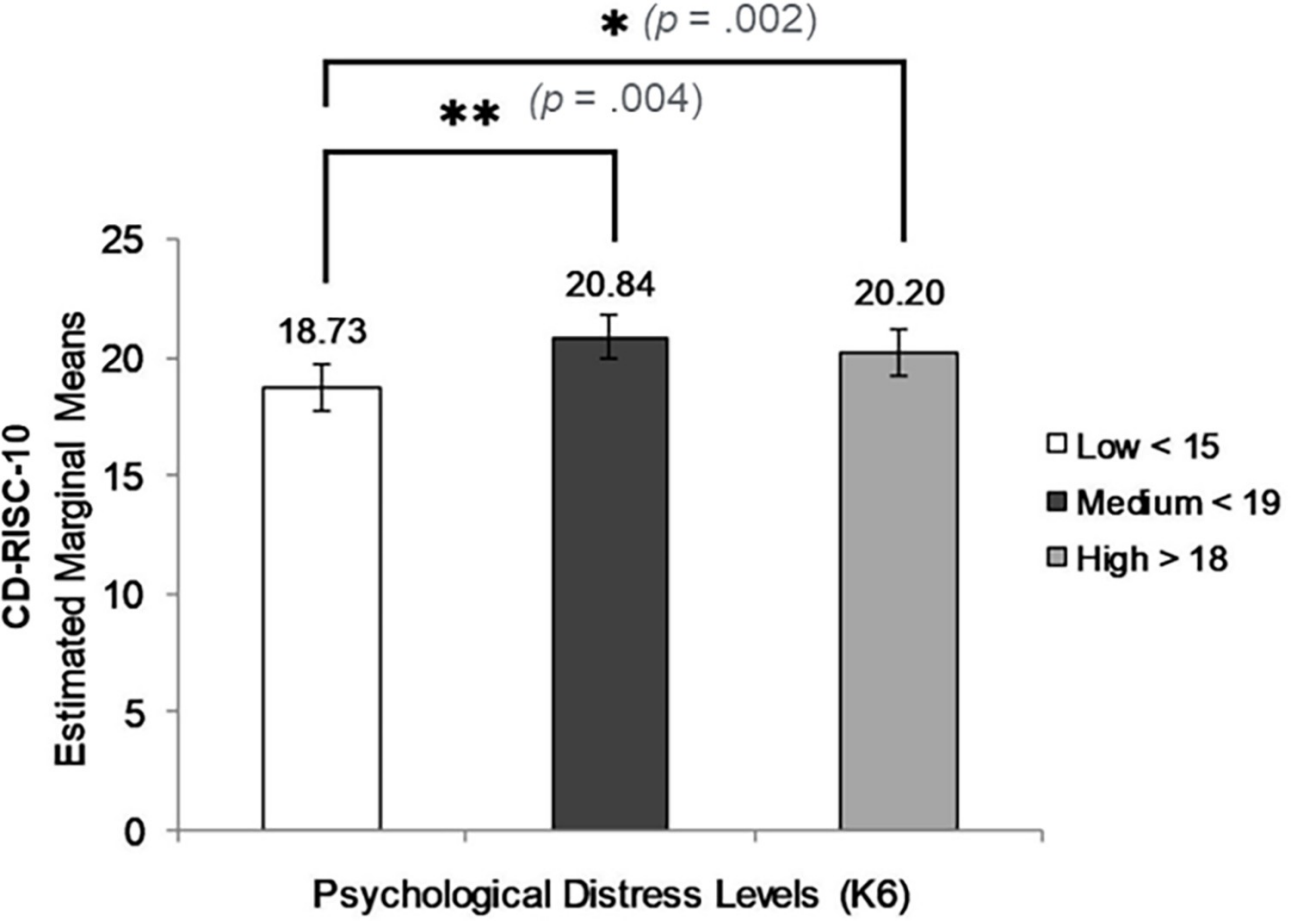
ANOVA results: CD-RISC-10 scores by level of psychological distress (K6).

ANOVA was used to test for the association between resilience and levels of psychological distress and emotional-behavioural difficulties. Students with higher levels of psychological distress (K6 scores) had significantly higher psychological resilience (CD-RISC-10 scores) than students with the lowest level of distress (*F* (2, 516) = 4.96, *p* = .007, η_p_^2^ = .019, power (1-β) = .81) (Fig 2). Error bars represent 95% confidence intervals.

**Fig 3:**
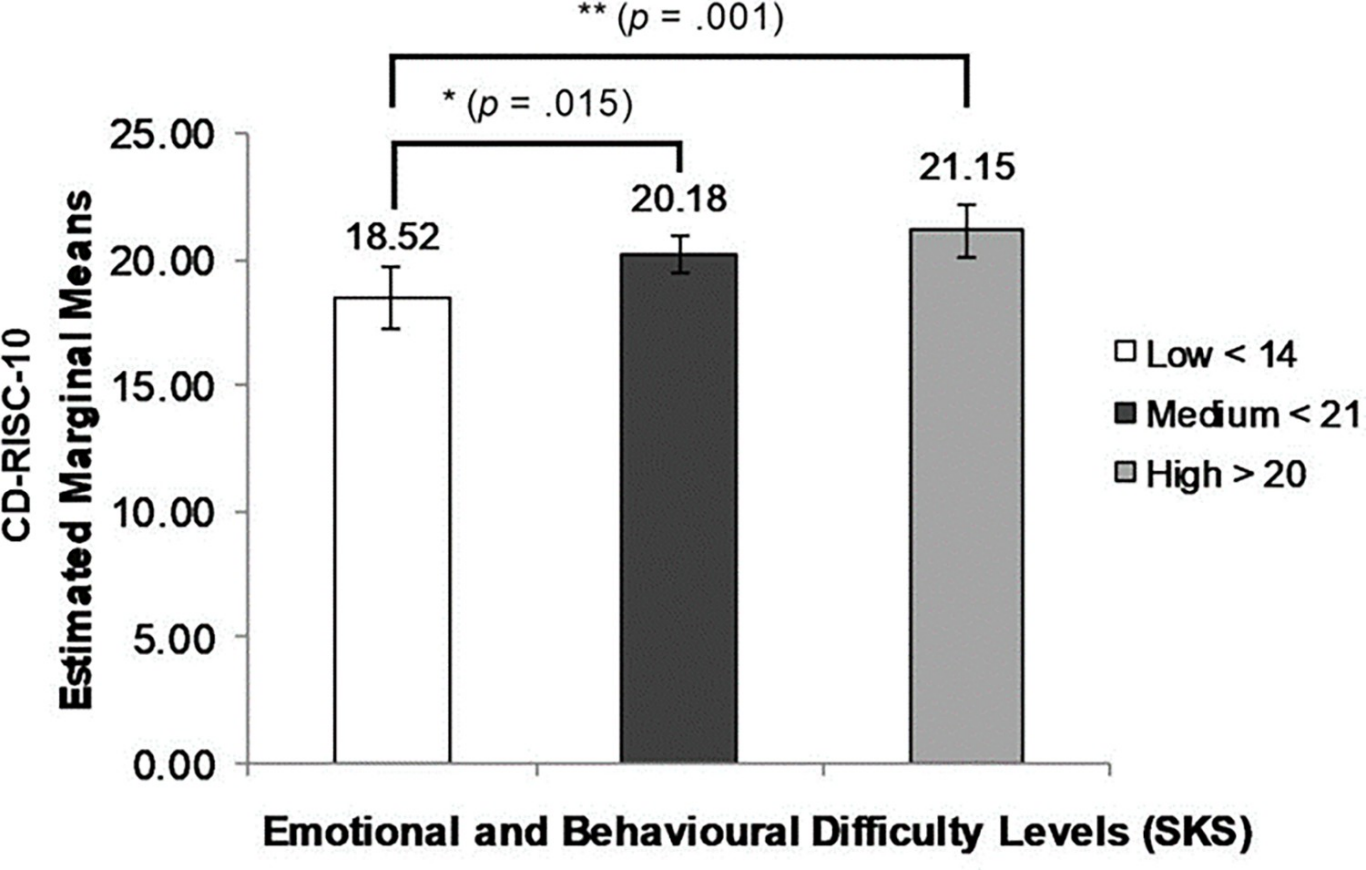
ANOVA results: CD-RISC-10 resiliency scores by level of emotional and behavioural difficulty (SKS).

Similarly, students with higher levels of emotional and behavioural problems (SKS) had higher CD-RISC-10 scores (*F* (2, 516) = 6.51, *p* = .002, η_p_^2^ = .025, power (1-β) = .91) (Fig 3).

**Fig 4:**
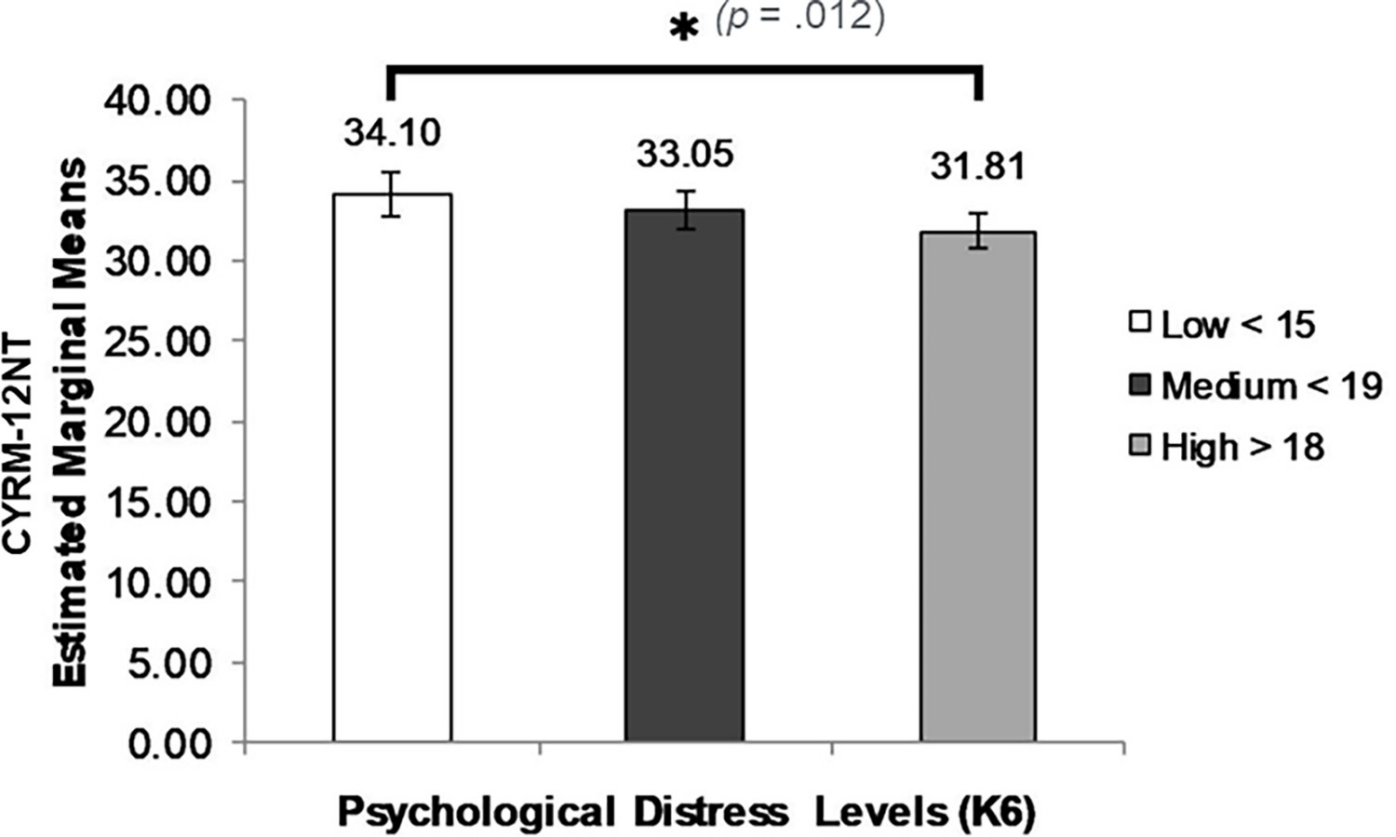
ANOVA results: CYRM-12NT scores by level of psychological distress (K6).

By contrast, the expected inverse association between socio-cultural resilience and psychological distress was significant: students with the lowest level of psychological distress (K6 scores) showed significantly higher CYRM-12NT scores (*F* (2, 516) = 3.15, *p* = .043, η_p_^2^ = .012, power (1-β) = .60) (Fig 4).

The expected inverse association between socio-cultural resilience (CYRM-12NT) and level of emotional and behavioural difficulty (SKS) was not significant for the whole sample.

#### Criterion validity, psychological resilience: CD-RISC-10

After hierarchical multiple regression analysis, a significant positive coefficient was observed with psychological resilience (CD-RISC-10) for K6, psychological distress (Table 6). A one-unit increase in resilience was associated with a 0.07 unit increase in distress as estimated with the unstandardised coefficient, *b*, when student age, sex, and life stressors were controlled, explaining 9% of the total variance in distress. The other significant explanatory variable, number of life stressors, was more strongly associated with psychological distress than with psychological resilience. There was a 0.26 standard deviation increase in psychological distress (K6 scores) for each standard deviation of life stressors, compared with psychological resilience (CD-RISC-10) for which one standard deviation predicted a 0.11 standard deviation increase in scores, as indicated in the standardised coefficient, β.

**Table 6:** Multiple regression analysis results explaining K6 and SKS with CD-RISC-10 and control variables.

Measure	Variable	*b*	95% CI for *b*		*SE* (*b*)	β	*p*
			*LL*	*UL*			
K6	Age	-0.01	-0.28	0.28	0.14	0	.968
	Sex	0.05	-0.68	0.75	0.37	.01	.892
	LS	0.63	0.40	0.87	0.12	.26	.001
	CD-RISC-10	0.07	0.004	0.13	0.03	.11	.035
SKS	Age	-0.08	-0.44	0.32	0.20	-.01	.701
	Sex	0.71	-0.42	1.79	0.55	.05	.201
	LS	1.45	1.13	1.77	0.16	.38	.001
	CD-RISC-10	0.14	0.05	0.22	0.05	.14	.004

*Note*: *N* = 516. CI: Confidence Interval; LL: Lower Limit; UL: Upper Limit.

When the regression was run by life stress level, of the three groups of reported life stressors– 0–2 (low); 3–4 (medium); 5–8 (high)—the association between scores for psychological resilience (CD-RISC-10) and for psychological distress (K6) was significant only in students who reported 0–2 stressors (See S4 Table). Among the students with 0–2 life stressors, each unit of psychological resilience suggested a 0.23 unit increase in psychological distress, explaining 5.3% of variance in K6 scores. No associations were significant in the groups with medium and high life stressors (S4 Table).

Emotional and behavioural difficulties (SKS scores) were also positively associated with psychological resilience (CD-RISC-10) scores: each unit of psychological resilience suggested a 0.14 unit increase in emotional and behavioural difficulties (Table 6). The model explained 18.5% of the total variance in SKS scores when student age, sex, and life stressors were taken into account. For life stress levels, only students with 0–2 life stressors were significantly more likely to have higher emotional and behavioural difficulties, with a 0.29 increase for each unit of psychological resilience explaining 10.4% variance of SKS (*p* < .001) while students with medium and high numbers of life stressors did not show any associations between resilience and difficulties in emotion and behaviour (S5 Table).

#### Criterion validity, socio-cultural resilience: CYRM-12NT

Associations between socio-cultural resilience (CYRM-12NT) and psychological distress (K6; *p* = .156) for the whole sample were not significant when students’ age, sex, and self-reported life stressors were controlled in multiple regression analysis. However, as presented in Table 7, school level analysis showed some significant results.

**Table 7:** Multiple regression analysis results by school explaining K6 with CYRM-12NT and control variables.

School	Variable	*B*	95% CI for *b* *LL UL*		*SE* (*b*)	Β	*P*
1	Age	-0.77	-1.50	-0.08	0.36	-.15	.032
*n* = 147	Sex	-0.12	-1.47	1.24	0.70	-.01	.867
	LS	0.63	0.22	1.03	0.21	.28	.003
	CYRM-12NT	-0.02	-0.12	0.07	0.05	-.04	.600
2	Age	0.40	-2.06	2.35	1.13	.06	.637
*n* = 27	Sex	0.03	-3.55	2.92	1.77	0	.983
	LS	2.11	1.09	3.09	0.50	.68	.001
	CYRM-12NT	-0.05	-0.32	0.19	0.13	-.08	.679
3	Age	0.63	-0.17	1.40	0.39	.16	.115
*n* = 83	Sex	-0.59	-2.41	1.24	0.93	-.07	.536
	LS	0.96	0.37	1.48	0.28	.37	.002
	CYRM-12NT	-0.10	-0.24	0.03	0.07	-.17	.122
4	Age	-0.32	-1.22	0.54	0.44	-.06	.469
*n* = 109	Sex	0.28	-1.38	1.98	0.84	.03	.726
	LS	0.57	0.13	1.08	0.24	.24	.016
	CYRM-12NT	-0.14	-0.26	-0.002	0.07	-.21	.041
5	Age	0.16	-0.21	0.53	0.19	.06	.400
*n* = 154	Sex	0.26	-1.18	1.65	0.72	.03	.717
	LS	0.43	-0.04	0.84	0.22	.17	.060
	CYRM-12NT	0.03	-0.07	0.12	0.05	.07	.516

*Note*. LL: Lower Limit; UL: Upper Limit.

In School 4, a significant decrease in psychological distress (K6) was associated with higher socio-cultural resilience (CYRM-12NT) scores (*p* = .041; *n* = 109) suggesting a 0.14 unit lower psychological distress score for each unit of socio-cultural resilience. The model explained 9.8% of the total variance in K6 for School 4. In School 3, although the negative coefficient was not significant (*p* = .122; *n* = 83), a 0.10 unit decrease in psychological distress was suggested for each unit of socio-cultural resilience, explaining 22.6% of the variance in psychological distress. Associations between socio-cultural resilience and psychological distress in other schools were not significant.

When the associations between socio-cultural resilience (CYRM-12NT) and psychological distress (K6) scores were tested by life stress levels, students who reported 3–4 stressors, (the medium level) showed a non-significant negative coefficient (*p* = .086, *n* = 218) albeit with a 0.12 unit decrease in psychological distress for each unit of socio-cultural resilience. The model explained 5% of the total variance in psychological distress.

In terms of students’ emotional and behavioural difficulties (SKS), associations between socio-cultural resilience (CYRM-12NT) and emotional and behavioural difficulties for the whole sample were not significant when students’ age, sex, and self-reported life stressors were controlled in multiple regression analysis (*p* = .419). However, in School 4, there was a significant negative association suggesting a .25 unit decrease in emotional and behavioural difficulties for each unit of socio-cultural resilience (*p* = .006, *n* = 106). The model explained 20.3% of the total variance in SKS scores in School 4.

In the whole sample, for students who reported life stressors in the medium range, (3–4 stressors, *n* = 218), a negative association between CYRM-12NT and SKS was not significant (*p* = .117). A non-significant 0.11 unit decrease in SKS was estimated by each unit of CYRM-12NT, explaining 5.1% of the total variance in SKS.

## Discussion

The present study examined the reliability and validity of two widely used resilience measures of psychological resilience, CD-RISC-10, and socio-cultural resilience, CYRM-12NT, using baseline data from 520 students in grades 7–10 at five schools implementing *Skills for Life* over three years. The analysis included two self-report criterion measures, the K6, a measure of general psychological distress, and the SKS, a measure of emotional and behavioural difficulties, along with life stressors experienced during the past month.

### Validity and reliability of the resilience measures

The results of confirmatory factor analysis (CFA) provided evidence for the construct validity of the two measures trialled. For CD-RISC-10, CFA supported a single-factor model of psychological resilience. The factor loadings (the correlation coefficient between each variable and the latent resilience factor) of the 10 indicator items ranged from .28 - .42. These were consistently lower than factor loadings (from .44 to .74) from the original validation of the CD-RISC-10 [25]. Supplementary analysis, treating Likert scale scores as ordered categorical variables, confirmed good model fit, with slightly higher factor loadings than in the main analysis. The results indicate that CD-RISC-10 showed acceptable construct validity in the present sample.

The CYRM-12NT measures external resources, including supportive family, peer, school and community relationships as sources of “socio-cultural resilience” [31]. CFA for CYRM-12NT supported a one-factor model, with factor loadings ranging from 0.336–0.605. This range is close to findings reported in other studies. In the original validation of the CYRM-12 with Canadian adolescents, aged from 14–22 years (*M* = 18; *SD* = 2.02), loadings for all items ranged from 0.388 to 0.844 [31]. These results were also confirmed in supplementary analysis treating scores as ordered categorical variables. Overall, the CYRM-12NT presented acceptable construct validity in the present sample, comparable with results elsewhere [32].

#### Internal consistency reliability of resilience measures

For CYRM-12NT, Cronbach’s alpha was acceptable at 0.74 for the overall sample. For CD-RISC-10, Cronbach’s alpha was 0.62 for the whole sample and as low as 0.53 in School 3. Taken together with the low factor loadings for CD-RISC-10, low internal consistency appears to indicate poor interrelatedness between items. However, this may not necessarily suggest construct heterogeneity [45]. It is likely related to a combination of age, linguistic and socio-cultural characteristics of the sample which affect consistency of student responses.

There were variations in Cronbach’s alpha between scales. The individual-psychological scales such as CD-RISC-10 and K6 showed lower reliability than the socio-cultural (CYRM-12NT) and behavioural (SKS) scales, suggesting that these students are more comfortable with questions about external, observable phenomena, such as relationships and behaviour than with questions about their inner states, feelings or attitudes. Cronbach’s alpha for the K6 was well below acceptable levels, at 0.50 overall and a low of 0.39 in School 5. Difficulty in use of the K6 with adolescents has elsewhere led to augmentation of the scale with items addressing emotional and behavioural symptoms [46].

There were also differences in reliability between schools. Cronbach’s alpha for the School 5 sample was markedly lower than other schools for CD-RISC-10, K6 and, to a lesser extent SKS. This may be explained by community characteristics. School 5 is located in a remote community with a history of low school attendance which has only begun to rise in recent years. In responses to individual CYRM-12NT items, 32% of students in School 5 responded that, “Getting an education is important to me” was “never true”, or only “a little of the time true”. For other schools, only 13% of students gave these responses.

These between-school differences suggest that cultural, linguistic and educational differences may influence responses to the questionnaires by students from these different locations. Although the language of instruction at all schools is English, 90% or more of students speak Aboriginal languages at home and in the community. These communities are internally diverse; they include first language speakers of multiple distinct Aboriginal languages, each language group having its own social and traditional community affiliations and histories of external engagement. Variable depth of positive engagement with formal education in the different communities is likely to influence students’ comprehension of and engagement with the questionnaires.

### Resilience, psychosocial risk and life stress

It was hypothesised that students’ level of psychological distress and emotional and behavioural difficulty when exposed to life stressors would be lower for those with higher measured resilience. However, criterion validity tests using cross-sectional variables of psychological distress (K6) and emotional and behavioural difficulties (SKS), produced differences between the two resilience measures. For psychological resilience, CD-RISC-10, there was an unexpected *positive* association between resilience score and levels of psychological distress (K6) and emotional-behavioural difficulty (SKS). Analysis of student subgroups by level of psychological distress showed significantly higher psychological resilience for students with K6 > 18. However, when adjusted for level of life stress, the positive relationship was statistically significant only for students reporting low numbers (0–2) of life stressors. This suggests that, for these youth, both psychological distress and behavioural symptoms need to be better understood in terms of their significance for positive adaptation to stress, rather than as directly indicative of potential for mental ill health, as has been the case in use of the K6 elsewhere [47].

A different pattern was observed for socio-cultural resilience (CYRM12-NT). There was a *negative* association between resilience scores and both psychological distress (K6) and emotional-behavioural problems (SKS). In one school, higher CYRM-12NT scores were significantly associated with lower levels of both psychological distress (K6) and emotional-behavioural difficulties (SKS), *p =* .*006*. In addition, for students with a medium level of life stress (3–4 stressors), higher socio-cultural resilience predicted lower psychological, emotional and behavioural risks (K6 and SKS scores).

#### Low resilience scores

Mean scores for psychological resilience, CD-RISC-10, at just under 20, appear to be low. In a review of studies using the CD-RISC-10 for a range of populations, scores typically ranged between the high 20s to low 30s for both general community samples and samples with specific health conditions [26]. Scores in the low twenties were only recorded in clinic counselling samples or samples exposed to significant trauma, with PTSD or depression: for example, a score of 20 was found among Japanese adolescents after exposure to a natural disaster [48]. By contrast, mean scores for socio-cultural resilience (CYRM-12NT) were not lower than reconstructed means of the original CYRM12 validation study (Table 3).

Comparatively low scores do not imply a lack of validity of the CD-RISC-10 as a measure of psychological resilience for this sample. They are likely to partly reflect low levels of psychological resilience in circumstances of adversity in families and communities which these young people feel are outside of their control. A proportion of these young people may have been exposed to early maltreatment and developmental difficulties [22]. However, low psychological resilience scores also suggest that individual resources are culturally less valued in these settings and may be perceived as secondary to and perhaps as less effective or relevant than external (social, familial) resources in dealing with the kinds of adversity faced by students in these communities.

#### Associations between resilience, adversity and distress

On average, students in these communities had recently experienced 3.4 life stressors. According to Luthar and others [49], resilience is not uni-dimensional, and individuals may be able to function even when manifesting high levels of psychological distress. Individuals experiencing distress and manifesting behavioural symptoms may therefore be resilient [50]. The positive association between psychological resilience and psychological distress and behavioural symptoms (K6 and SKS scores) suggests that exposure to these stressors activates a sense of capacity to cope while at the same time contributing to elevated distress. while Elevated behavioural and emotional symptoms may be associated with styles of adaptation to stressors. Emotional and behavioural symptoms may in fact reflect active coping in stressful familial and social environments. As a measure of psychological resilience, CD-RISC-10 appears to describe awareness of challenges faced and a sense of need, if not ability to cope with them [15].

For this sample of remote youth, the association between higher psychological distress and behavioural symptoms and higher psychological resilience was significant for those with low, but not for those with high numbers of life stressors. Higher CD-RISC-10 scores may reflect confidence in coping with a moderate degree of life stress causing distress and behavioural symptoms but do not identify characteristics and resources needed to cope with the higher levels of stress encountered by many young people in this sample.

The CYRM-12NT aims to measure the individual’s assessment of external relationships as sources of strength and support. These dimensions of resilience are both important and recognisable to this population of students, and, when present, contribute to reduced levels of psychosocial risk and distress. The negative association between CYRM-12NT scores and psychological distress (K6) and emotional-behavioural symptoms (SKS) overall, and more markedly in two schools, bears out this conclusion. Most of the life stressors indicated by the students are inherently stressors within the ecology of relationships at home and in the community among peers: family fighting, bullying, drinking alcohol, suicidal behaviour, police intervention at home, loss of someone close, etc. It is to be expected that higher expectations of and greater capacity to engage support within family, peers and community would be associated with lower levels of distress and emotional-behavioural difficulty.

#### Cultural dimensions of resilience

At first sight, the difference between the two measures is between the individual’s sense of self-efficacy and expectation of ability to cope, and assessment of external resources and expectations of support. Resilience research has frequently found that internal locus of control is associated with higher levels of resilience [51,52]. CD-RISC-10 items are consistent with a highly individualised, internal model of self-efficacy–and an implied model of self and capacity for self-appraisal independently of others. The CYRM-12NT items appear to better correspond with an external orientation of self to others and a sense of self mediated through relationships–a relational concept of self, and a sense of self-efficacy contingent on the availability of others. The dimension of culture remains invisible in the former conception of the individual self, insofar as it excludes relationship and connectedness in forms that are culturally recognisable within each context. The low scores for CD-RISC-10 may therefore in part reflect a developmental and cultural context in which individual self-efficacy or agency are more likely to be expressed in terms of interdependence with related others and in which challenge and adversity are experienced above all in terms of relationships with others. In terms of cultural self-understanding, it appears that for these young adolescents, individual agency is less valued than location in relationships when coping with adversity.

The different conceptions of resilience are also dependent on developmental age. Dependence on others is universally legitimate and expected of youth but in socially and culturally different ways. Individualism and internal control are normatively associated with age and age-appropriate expectations of maturity, self-efficacy and independent responsibility. These are distinctively embodied in Western formal education, which has only in recent decades sought to explicitly incorporate a developmentally informed model for social and emotional learning that encompasses relational competencies. However, resilience in both senses–individual and socio-cultural or relational—needs to be interpreted for age, and against the background of life course-dependent culturally formed expectations.

### Limitations

This sample was accumulated in five remote schools over three years, rather than administered as a single cross-sectional study. Numbers are unequal between the schools, and low attending students are likely to be under-represented, thus limiting representativeness of the sample across all regions. Despite every effort to ensure consistency in the method of data-gathering in the schools by trained administrators, there may have been variation across different school and community environments. To better deal with these challenges, review and simplification of items and use of visual Likert scaling with questions on electronic tablet screens should be trialled.

## Conclusions

This study has demonstrated that measurement of dimensions of resilience through students’ self-report using standardised measures is feasible and that the two constructs, psychological resilience and socio-cultural resilience meet criteria for validity for this sample. However, questions remain concerning their predictive validity. These two measures imply different understandings of risk: the CD-RISC-10 implies a generalised notion of setbacks that are perceived as such by individuals; the CYRM-12NT implies an understanding of risks that are implicitly amenable to intervention or support by others—persons, family, school or community. Despite potential complementarity between these measures, the one ostensibly individual-psychological and the other external-relational, it is suggested that further development of these measures is required for evaluative purposes.

The model of psychological resilience implied by CD-RISC-10 is too narrow to capture the range of individual competencies and dispositions underpinning psychological resources among Aboriginal students from these remote communities. It does not differentiate capabilities which can help manage higher levels of adversity and distress. Others have noted that modification of this scale by augmentation with additional items may be justified [30]. The model of socio-cultural resilience implied by CYRM-12NT captures relevant orientation to valued resources and relationships and may discriminate the kinds of support that reduce distress and protect against risk in these communities. However, while appearing to have demonstrated adequate performance for use as a contextually relevant measure in remote settings, with its current structure and items, the CYRM-12NT has limitations. It may not do justice to the competencies needed to negotiate those resources consistent with culturally formed styles of communication and social interaction within these communities. These shape both behavioural expressions of distress and elicitation of supportive responses, for example through shows of anger and destructive behaviour including self-harm, as modelled both by youths and adults [53].

Between-school differences highlighted in this analysis may point to sources of variance across communities based on subtle community differences, as a recent multi-country meta-analysis has found for the longer version, the CYRM28 [33]. Given the complex interacting differences in traditional and contemporary cultures, beliefs and practices, formal education and literacy, family relationships and social conditions in remote Aboriginal communities, there is a case for further attention to item development to improve the predictive validity of CYRM for these very remote communities, along lines conducted in a recent study in the middle east [34]. This will enable better identification of factors most able to contribute to resilient adaptation through social and emotional learning.

Further research into resilience among remote Aboriginal youth must investigate responses to risk in circumstances of high and ongoing life stress. Findings suggest that there is a need to further investigate the interaction between psychological distress and relevant emotional and behavioural symptoms and associated help-seeking behaviours. The life stressors identified here are almost certainly associated with elevated risk of adverse outcomes, including self-harm. However, it was not possible to differentiate either proximate from distal exposure to stressors, or acute from chronic experiences of life stress to better identify individual risk [22]. A more detailed investigation of developmental precursors of vulnerability, such as early neglect and maltreatment would help to identify which students and under what circumstances might be most at risk of the poorest outcomes [54].

Elders consulted during the development of *Skills for Life* emphatically stressed that awareness of emotions and emotional competencies are critically important for the ability of young people to negotiate social inclusion and build their strengths. That this was absent from Western school education was a matter of concern to them. Styles of interpersonal communication, self-regulation and social awareness are for these elders the primary terrain of Aboriginal culture. In light of their concern and of findings of this study, it is concluded that collaborative work to further develop and test tools to measure competencies that can lead to positive outcomes, is a priority.

## Supporting information

S1 Table CDRISC-10 CFA factor loadings calculated using RWLS estimator. (DOCX)

S2 Table CYRM12-NT CFA factor loadings calculated using RWLS estimator. (DOCX)

S3 Table One-way ANOVA: K6 and SKS by number of life stressors. (DOCX)

S4 Table Multiple regression analysis: Explaining K6 with CD-RISC-10 by life stress level. (DOCX)

S5 Table Multiple regression analysis: Explaining SKS with CD-RISC-10 by life stress level. (DOCX)
