# Supplementary material for: Validity and reliability of resiliency measures trialled for the evaluation of a preventative Resilience-promoting social-emotional curriculum for remote Aboriginal school students
Source: PLoS One. 2022 Jan 11;17(1):e0262406. doi: 10.1371/journal.pone.0262406 (PMC8752014; doi:10.1371/journal.pone.0262406)
Supplement: S1 Table — (DOCX) [file pone.0262406.s001.docx]

**S1 Table. Confirmatory Factor Analysis of the 10-item Connor-Davidson Resilience Scale (CD-RISC-10): Factor Loadings Estimated by a Robust Weighted Least Squares Estimator.**

| Item | Description | Factor  Loading |
| --- | --- | --- |
|  |  |  |
| 1 | I can handle it when changes happen | .40 |
| 2 | I can deal with whatever comes my way | .42 |
| 3 | I try to see the funny side when problems come up | .31 |
| 4 | Having to cope with stress can make me stronger | .33 |
| 5 | I tend to bounce back quickly after illness, injury or hard time | .45 |
| 6 | I believe I can achieve my goals even when things stand in my way | .44 |
| 7 | I can stay focused and think clearly under pressure | .42 |
| 8 | I don't give up easily when I fail | .43 |
| 9 | I think of myself as a strong person who can deal with difficulties and challenges in life | .42 |
| 10 | I can handle bad feelings like sadness, fear and anger | .45 |

Model fit: (χ^2^ (35, N = 520) = 59.55, *p* = .006; *CFI* = .94, *TLI* = .93, *RMSEA* = .04).
