# Supplementary material for: Validity and reliability of resiliency measures trialled for the evaluation of a preventative Resilience-promoting social-emotional curriculum for remote Aboriginal school students
Source: PLoS One. 2022 Jan 11;17(1):e0262406. doi: 10.1371/journal.pone.0262406 (PMC8752014; doi:10.1371/journal.pone.0262406)
Supplement: S2 Table — (DOCX) [file pone.0262406.s002.docx]

**S2 Table. Confirmatory Factor Analysis of the 12-item Child and Youth Resilience Measure (CYRM-12NT): Factor Loadings Estimated by a Robust Weighted Least Squares Estimator.**

| Item | Description | Factor Loading |
| --- | --- | --- |
|  |  |  |
| 1 | I have people who I look up to | .38 |
| 2 | Getting an education at school is important to me | .71 |
| 3 | My parents/caregivers know a lot about me | .52 |
| 4 | When I start things I always try to finish them | .43 |
| 6 | I know who or where I can go to in my community to get help | .52 |
| 7 | I feel that I belong at my school | .51 |
| 8 | My family will stand by me when I am having a hard time | .41 |
| 9 | I have friends who will stand by me when I am having a hard time | .46 |
| 10 | I am treated fairly in my community | .42 |
| 11 | In my community I am able to learn skills and knowledge that will help me in life | .58 |
| 12 | Culture in my family and community is important to me | .57 |
| 13^a^ | At my school I can learn skills that will help me get work | .65 |

*Note* ^a^: The newly developed item in the CYRM-12NT.

Model fit: (χ^2^ (53, N = 520) = 116.17, *p* < .001; *CFI* = .95, *TLI* = .94, *RMSEA* = .05).
