# Supplementary material for: Validity and reliability of resiliency measures trialled for the evaluation of a preventative Resilience-promoting social-emotional curriculum for remote Aboriginal school students
Source: PLoS One. 2022 Jan 11;17(1):e0262406. doi: 10.1371/journal.pone.0262406 (PMC8752014; doi:10.1371/journal.pone.0262406)
Supplement: S3 Table — (DOCX) [file pone.0262406.s003.docx]

**S3 Table. Means, Standard Deviations, and One-Way Analyses of Variance (ANOVA) in Psychological Distress (K6) and Emotional and Behavioural Difficulties (SKS) across the Total Number of Life Stressors**

| Number of Life Stressors/  ANOVA Statistics | Measures | | | |
| --- | --- | --- | --- | --- |
|  | Psychological Distress | | Emotional and Behavioural Difficulties (SKS) | |
|  | (K6) | |  |  |
|  | *M* | *SD* | *M* | *SD* |
| 0 | 8.19 | 5.33 | 11.71 | 7.14 |
| 1 | 7.56 | 4.89 | 11.22 | 6.46 |
| 2 | 8.05 | 4.18 | 13.17 | 6.12 |
| 3 | 9.24 | 4.39 | 15.29 | 6.46 |
| 4 | 10.77 | 3.55 | 17.53 | 5.93 |
| 5 | 11.18 | 3.73 | 19.36 | 5.70 |
| 6 | 10.32 | 4.31 | 19.03 | 5.76 |
| 7 | 11.83 | 4.72 | 20.42 | 7.13 |
| 8 | 11.71 | 4.50 | 19.43 | 11.19 |
| *F* (8,509) | 6.94*** | | 13.82*** | |
| η^2^ | .10 | | .18 | |

****p* < .001.
