# Supplementary material for: Validity and reliability of resiliency measures trialled for the evaluation of a preventative Resilience-promoting social-emotional curriculum for remote Aboriginal school students
Source: PLoS One. 2022 Jan 11;17(1):e0262406. doi: 10.1371/journal.pone.0262406 (PMC8752014; doi:10.1371/journal.pone.0262406)
Supplement: S4 Table — (DOCX) [file pone.0262406.s004.docx]

**S4 Table. Multiple Regression Analysis Results: Explaining K6 with CD-RISC-10 by Age, Sex and Life Stress Level**

| Life Stress Level | Variable | *b* | 95% CI for *b* | | *SE* (*b*) | β | *p* |
| --- | --- | --- | --- | --- | --- | --- | --- |
| (*R^2^*) |  |  | *LL* | *UL* |  |  |  |
| Low | Age | -0.52 | -0.55 | 0.42 | 0.25 | -.02 | .823 |
| (.05) | Sex | 0.62 | -0.61 | 2.05 | 0.70 | .07 | .382 |
|  | CD-RISC-10 | **0.15** | **0.03** | **0.27** | **0.06** | **.23** | **.019** |
| Medium | Age | 0.09 | -0.32 | 0.56 | 0.22 | .03 | .646 |
| (.001) | Sex | 0.10 | -0.92 | 1.10 | 0.52 | .01 | .849 |
|  | CD-RISC-10 | 0.05 | -0.06 | 0.15 | 0.05 | .07 | .392 |
| High | Age | -0.16 | -0.64 | 0.36 | 0.26 | -.05 | .369 |
| (.007) | Sex | -0.55 | -1.93 | 0.88 | 0.73 | -.07 | .883 |
|  | CD-RISC-10 | 0.01 | -0.12 | 0.13 | 0.06 | .02 | .132 |
| *Note.* Low: Students with 0-2 responses to the Life Stressors Checklist (*n* = 169); Medium: Students with 3-4 responses (*n* = 218); High: Students with 5-8 responses (*n* = 129); K6: The Kessler 6 Mental Health Scale; CD-RISC-10: The Connor-Davidson Resilience Scale; CI: Confidence Interval; LL: Lower Limit; UL: Upper Limit. | | | | | | | |
