# Supplementary material for: Validity and reliability of resiliency measures trialled for the evaluation of a preventative Resilience-promoting social-emotional curriculum for remote Aboriginal school students
Source: PLoS One. 2022 Jan 11;17(1):e0262406. doi: 10.1371/journal.pone.0262406 (PMC8752014; doi:10.1371/journal.pone.0262406)
Supplement: S5 Table — (DOCX) [file pone.0262406.s005.docx]

**S5 Table. Multiple Regression Analysis Results: Explaining SKS with CD-RISC-10 by Age, Sex and Life Stress Level**

| Life Stress Level | Variable | *b* | 95% CI for *b* | | *SE* (*b*) | β | *p* |
| --- | --- | --- | --- | --- | --- | --- | --- |
| (*R^2^*) |  |  | *LL* | *UL* |  |  |  |
| Low | Age | -.49 | -1.17 | .23 | .35 | -.10 | .165 |
| (.10) | Sex | 1.75 | .03 | 3.63 | .88 | .14 | .060 |
|  | CD-RISC-10 | **.27** | **.13** | **.39** | **.06** | **.29** | **.002** |
| Medium | Age | .54 | -.03 | 1.21 | .32 | .11 | .089 |
| (.02) | Sex | -.44 | -2.15 | 1.18 | .85 | -.04 | .576 |
|  | CD-RISC-10 | .08 | -.07 | .23 | .07 | .09 | .226 |
| High | Age | -.61 | -1.42 | .24 | .42 | -.117 | .142 |
| (.04) | Sex | 1.58 | -.57 | 4.08 | 1.17 | .125 | .182 |
|  | CD-RISC-10 | .07 | -.15 | .24 | .10 | .064 | .531 |
| *Note.* Low: Students with 0-2 responses to the Life Stressors Checklist (*n* = 169); Medium: Students with 3-4 responses (*n* = 218); High: Students with 5-8 responses (*n* = 129); SKS: The Strong Kids Symptoms Scale; CD-RISC-10: The Connor-Davidson Resilience Scale; CI: Confidence Interval; LL: Lower Limit; UL: Upper Limit. | | | | | | | |
